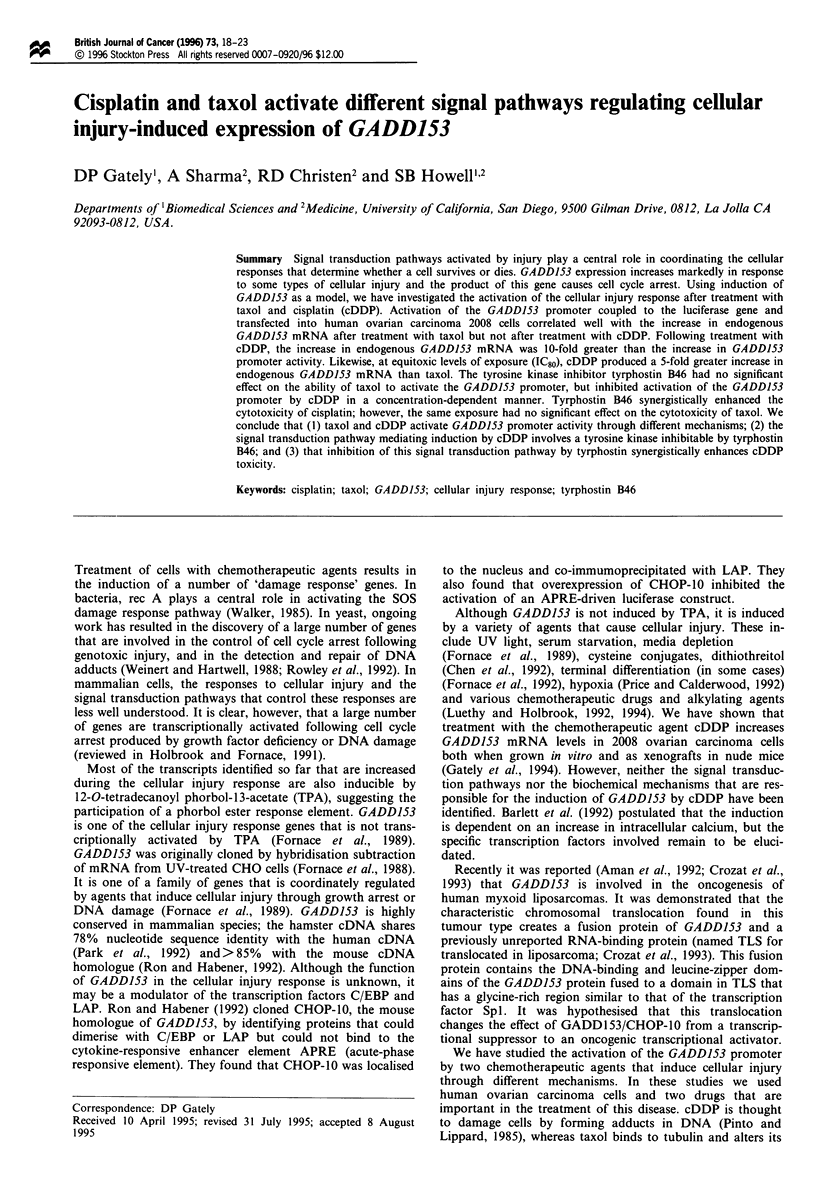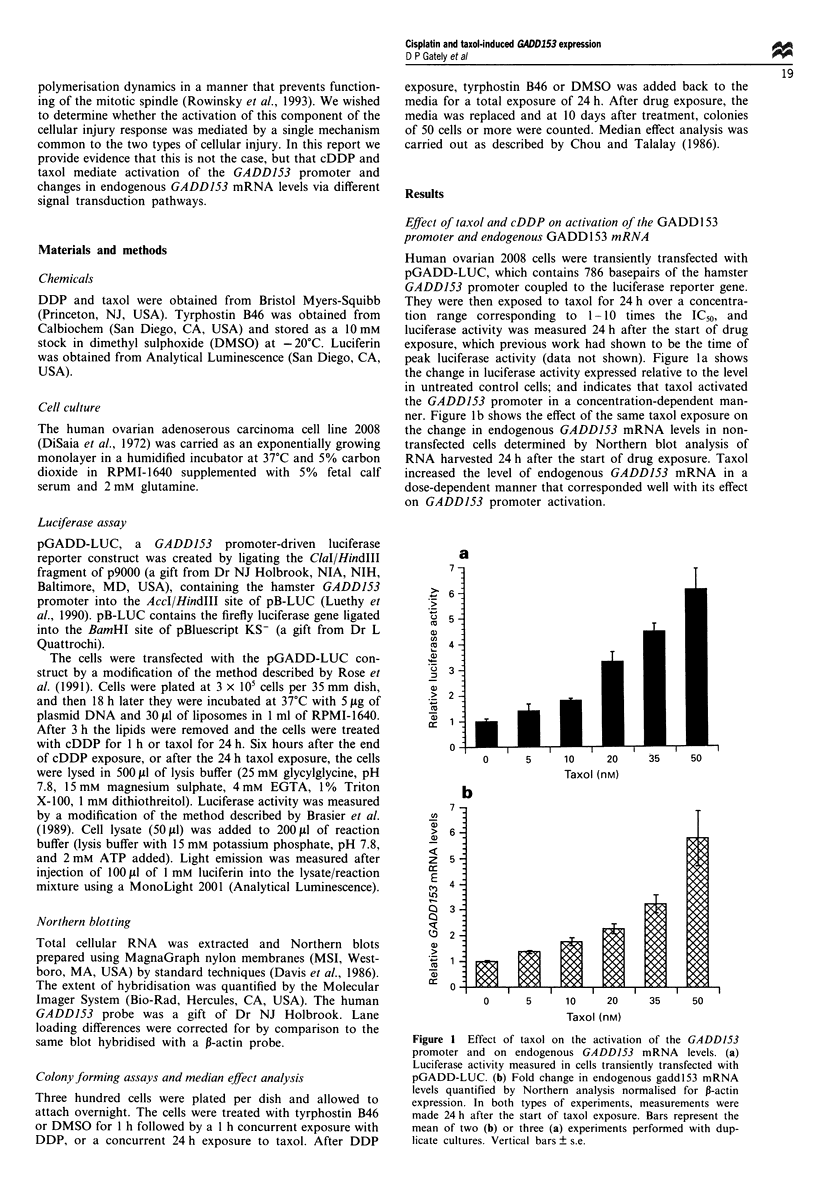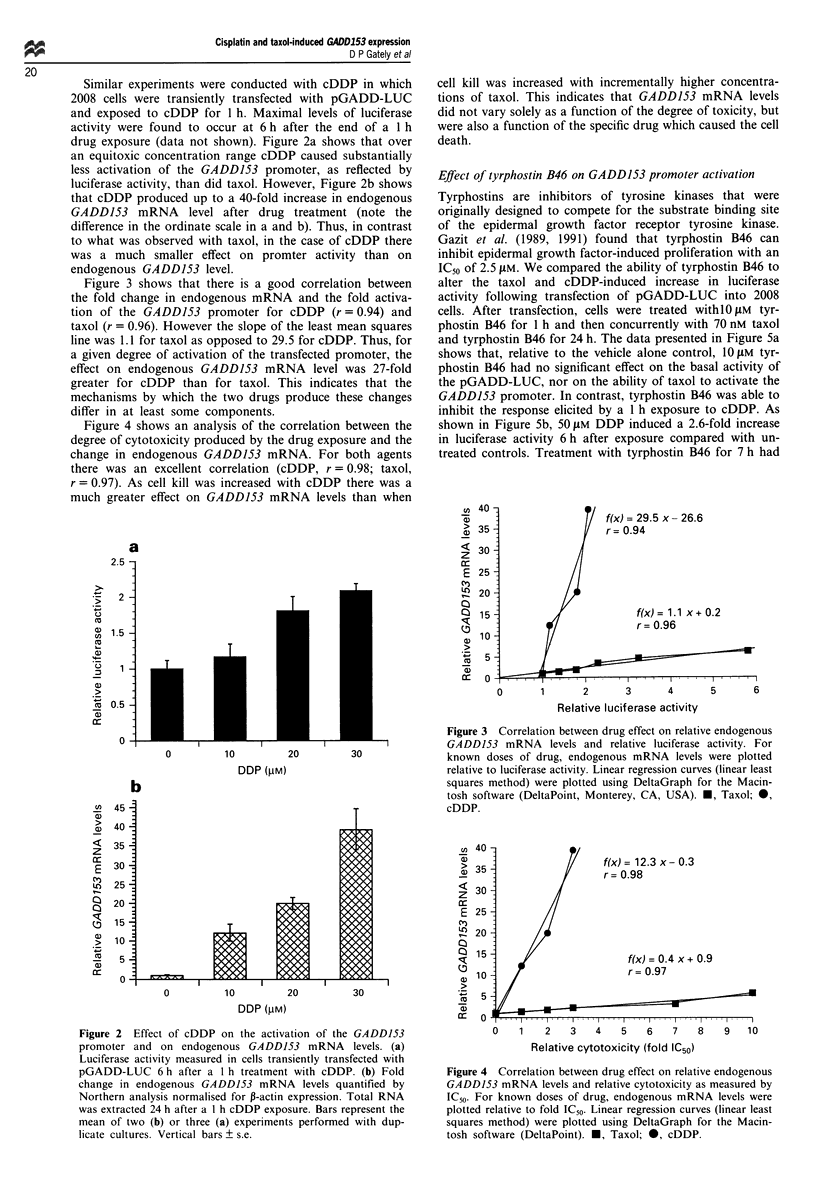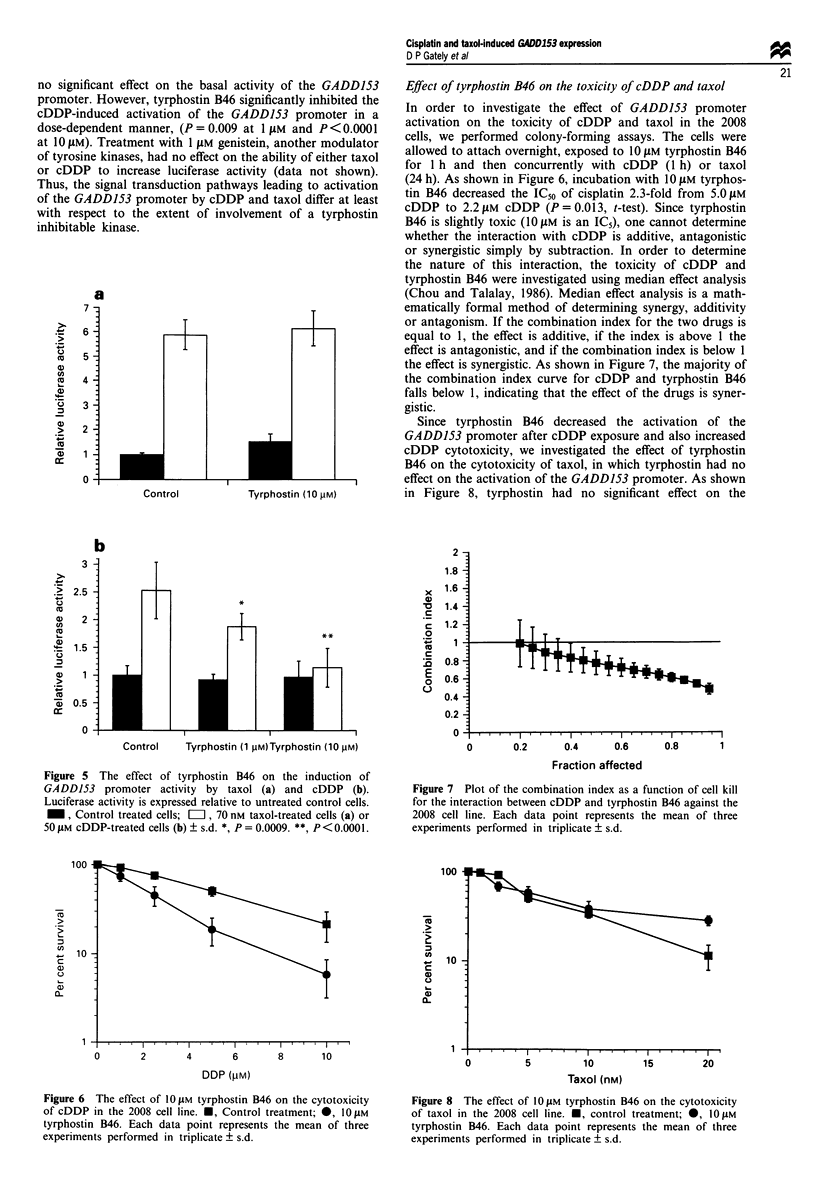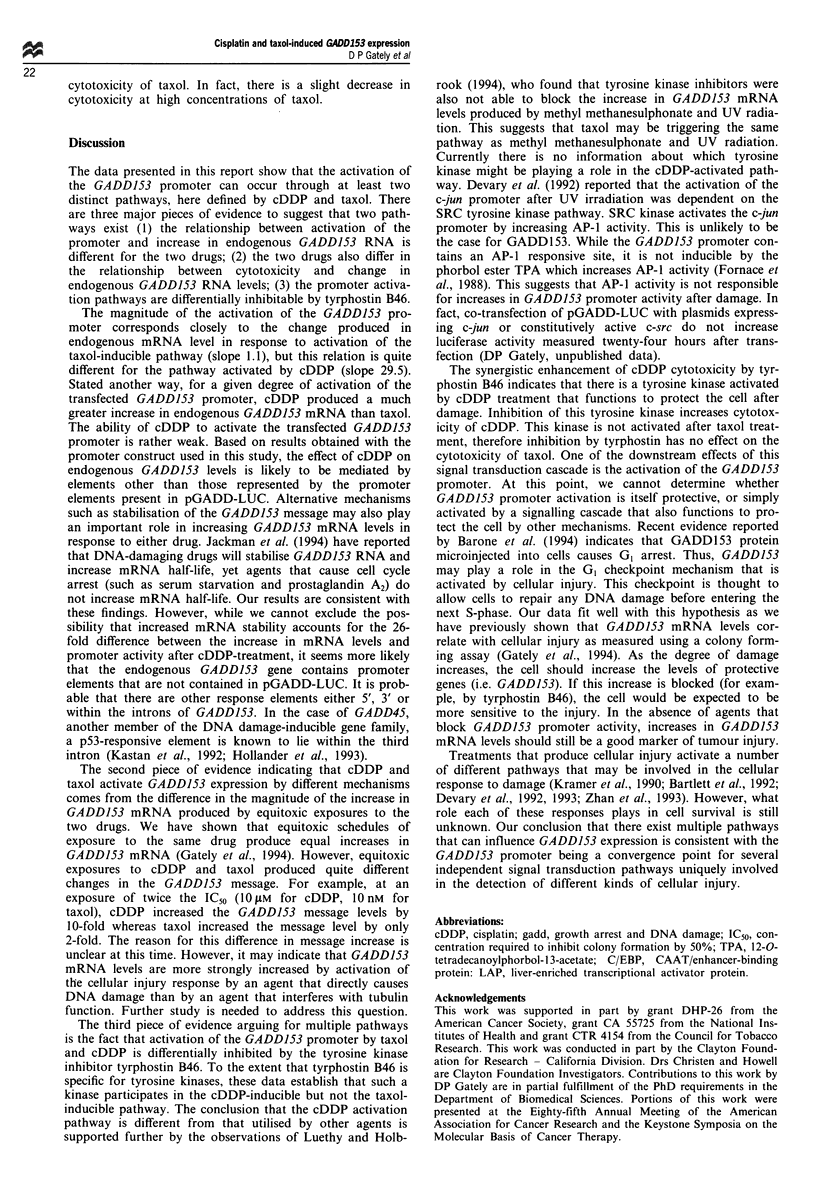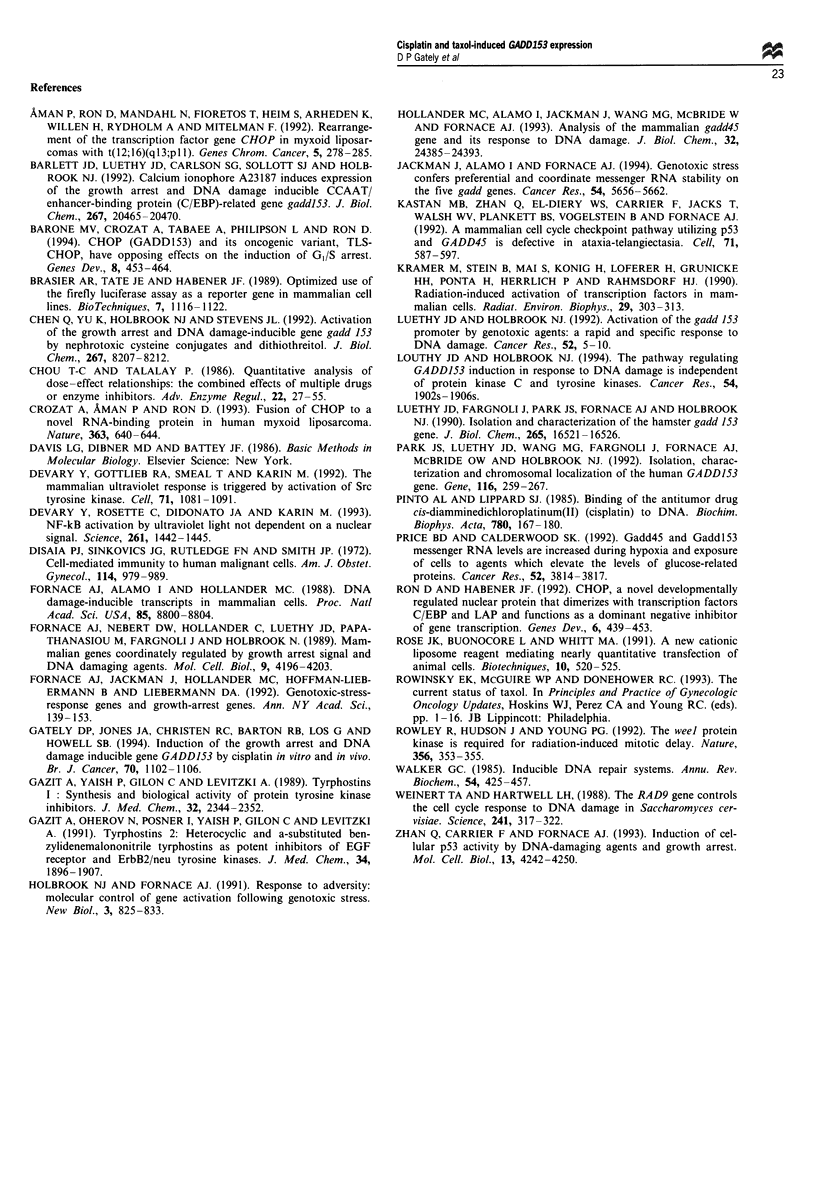# Cisplatin and taxol activate different signal pathways regulating cellular injury-induced expression of GADD153.

**DOI:** 10.1038/bjc.1996.4

**Published:** 1996-01

**Authors:** D. P. Gately, A. Sharma, R. D. Christen, S. B. Howell

**Affiliations:** Department of Biomedical Sciences, University of California, San Diego, La Jolla 92093-0812, USA.

## Abstract

Signal transduction pathways activated by injury play a central role in coordinating the cellular responses that determine whether a cell survives or dies. GADD153 expression increases markedly in response to some types of cellular injury and the product of this gene causes cell cycle arrest. Using induction of GADD153 as a model, we have investigated the activation of the cellular injury response after treatment with taxol and cisplatin (cDDP). Activation of the GADD153 promoter coupled to the luciferase gene and transfected into human ovarian carcinoma 2008 cells correlated well with the increase in endogenous GADD153 mRNA after treatment with taxol but not after treatment with cDDP. Following treatment with cDDP, the increase in endogenous GADD153 mRNA was 10-fold greater than the increase in GADD153 promoter activity. Likewise, at equitoxic levels of exposure (IC80), cDDP produced a 5-fold greater increase in endogenous GADD153 mRNA than taxol. The tyrosine kinase inhibitor tyrophostin B46 had no significant effect on the ability of taxol to activate the GADD153 promoter, but inhibited activation of the GADD153 promoter by cDDP in a concentration-dependent manner. Tyrphostin B46 synergistically enhanced the cytotoxicity of cisplatin; however, the same exposure had no significant effect on the cytotoxicity of taxol. We conclude that (1) taxol and cDDP activate GADD153 promoter activity through different mechanisms; (2) the signal transduction pathway mediating induction by cDDP involves a tyrosine kinase inhibitable by tyrphostin B46; and (3) that inhibition of this signal transduction pathway by tyrphostin synergistically enhances cDDP toxicity.


British Journal of Cancer (1996) 73, 18-23
© 1996 Stockton Press All rights reserved 0007-0920/96 $12.00

### Cisplatin and taxol activate different signal pathways regulating cellular injury-induced expression of *GADD153*

DP Gately[1], A Sharma[2], RD Christen[2] and SB Howell[1,2]

*Departments of [1]Biomedical Sciences and [2]Medicine, University of California, San Diego, 9500 Gilman Drive, 0812, La Jolla CA 92093-0812, USA.*

**Summary** Signal transduction pathways activated by injury play a central role in coordinating the cellular responses that determine whether a cell survives or dies. *GADD153* expression increases markedly in response to some types of cellular injury and the product of this gene causes cell cycle arrest. Using induction of *GADD153* as a model, we have investigated the activation of the cellular injury response after treatment with taxol and cisplatin (cDDP). Activation of the *GADD153* promoter coupled to the luciferase gene and transfected into human ovarian carcinoma 2008 cells correlated well with the increase in endogenous *GADD153* mRNA after treatment with taxol but not after treatment with cDDP. Following treatment with cDDP, the increase in endogenous *GADD153* mRNA was 10-fold greater than the increase in *GADD153* promoter activity. Likewise, at equitoxic levels of exposure ($IC_{80}$), cDDP produced a 5-fold greater increase in endogenous *GADD153* mRNA than taxol. The tyrosine kinase inhibitor tyrphostin B46 had no significant effect on the ability of taxol to activate the *GADD153* promoter, but inhibited activation of the *GADD153* promoter by cDDP in a concentration-dependent manner. Tyrphostin B46 synergistically enhanced the cytotoxicity of cisplatin; however, the same exposure had no significant effect on the cytotoxicity of taxol. We conclude that (1) taxol and cDDP activate *GADD153* promoter activity through different mechanisms; (2) the signal transduction pathway mediating induction by cDDP involves a tyrosine kinase inhibitable by tyrphostin B46; and (3) that inhibition of this signal transduction pathway by tyrphostin synergistically enhances cDDP toxicity.

**Keywords:** cisplatin; taxol; *GADD153*; cellular injury response; tyrphostin B46

Treatment of cells with chemotherapeutic agents results in the induction of a number of 'damage response' genes. In bacteria, rec A plays a central role in activating the SOS damage response pathway (Walker, 1985). In yeast, ongoing work has resulted in the discovery of a large number of genes that are involved in the control of cell cycle arrest following genotoxic injury, and in the detection and repair of DNA adducts (Weinert and Hartwell, 1988; Rowley *et al.*, 1992). In mammalian cells, the responses to cellular injury and the signal transduction pathways that control these responses are less well understood. It is clear, however, that a large number of genes are transcriptionally activated following cell cycle arrest produced by growth factor deficiency or DNA damage (reviewed in Holbrook and Fornace, 1991).

Most of the transcripts identified so far that are increased during the cellular injury response are also inducible by 12-*O*-tetradecanoyl phorbol-13-acetate (TPA), suggesting the participation of a phorbol ester response element. *GADD153* is one of the cellular injury response genes that is not transcriptionally activated by TPA (Fornace *et al.*, 1989). *GADD153* was originally cloned by hybridisation subtraction of mRNA from UV-treated CHO cells (Fornace *et al.*, 1988). It is one of a family of genes that is coordinately regulated by agents that induce cellular injury through growth arrest or DNA damage (Fornace *et al.*, 1989). *GADD153* is highly conserved in mammalian species; the hamster cDNA shares 78% nucleotide sequence identity with the human cDNA (Park *et al.*, 1992) and >85% with the mouse cDNA homologue (Ron and Habener, 1992). Although the function of *GADD153* in the cellular injury response is unknown, it may be a modulator of the transcription factors C/EBP and LAP. Ron and Habener (1992) cloned CHOP-10, the mouse homologue of *GADD153*, by identifying proteins that could dimerise with C/EBP or LAP but could not bind to the cytokine-responsive enhancer element APRE (acute-phase responsive element). They found that CHOP-10 was localised to the nucleus and co-immumoprecipitated with LAP. They also found that overexpression of CHOP-10 inhibited the activation of an APRE-driven luciferase construct.

Although *GADD153* is not induced by TPA, it is induced by a variety of agents that cause cellular injury. These include UV light, serum starvation, media depletion (Fornace *et al.*, 1989), cysteine conjugates, dithiothreitol (Chen *et al.*, 1992), terminal differentiation (in some cases) (Fornace *et al.*, 1992), hypoxia (Price and Calderwood, 1992) and various chemotherapeutic drugs and alkylating agents (Luethy and Holbrook, 1992, 1994). We have shown that treatment with the chemotherapeutic agent cDDP increases *GADD153* mRNA levels in 2008 ovarian carcinoma cells both when grown *in vitro* and as xenografts in nude mice (Gately *et al.*, 1994). However, neither the signal transduction pathways nor the biochemical mechanisms that are responsible for the induction of *GADD153* by cDDP have been identified. Barlett *et al.* (1992) postulated that the induction is dependent on an increase in intracellular calcium, but the specific transcription factors involved remain to be elucidated.

Recently it was reported (Aman *et al.*, 1992; Crozat *et al.*, 1993) that *GADD153* is involved in the oncogenesis of human myxoid liposarcomas. It was demonstrated that the characteristic chromosomal translocation found in this tumour type creates a fusion protein of *GADD153* and a previously unreported RNA-binding protein (named TLS for translocated in liposarcoma; Crozat *et al.*, 1993). This fusion protein contains the DNA-binding and leucine-zipper domains of the *GADD153* protein fused to a domain in TLS that has a glycine-rich region similar to that of the transcription factor Sp1. It was hypothesised that this translocation changes the effect of GADD153/CHOP-10 from a transcriptional suppressor to an oncogenic transcriptional activator.

We have studied the activation of the *GADD153* promoter by two chemotherapeutic agents that induce cellular injury through different mechanisms. In these studies we used human ovarian carcinoma cells and two drugs that are important in the treatment of this disease. cDDP is thought to damage cells by forming adducts in DNA (Pinto and Lippard, 1985), whereas taxol binds to tubulin and alters its

Correspondence: DP Gately
Received 10 April 1995; revised 31 July 1995; accepted 8 August 1995

polymerisation dynamics in a manner that prevents functioning of the mitotic spindle (Rowinsky *et al.*, 1993). We wished to determine whether the activation of this component of the cellular injury response was mediated by a single mechanism common to the two types of cellular injury. In this report we provide evidence that this is not the case, but that cDDP and taxol mediate activation of the *GADD153* promoter and changes in endogenous *GADD153* mRNA levels via different signal transduction pathways.

### Materials and methods

### *Chemicals*

DDP and taxol were obtained from Bristol Myers-Squibb (Princeton, NJ, USA). Tyrphostin B46 was obtained from Calbiochem (San Diego, CA, USA) and stored as a 10 mM stock in dimethyl sulphoxide (DMSO) at −20°C. Luciferin was obtained from Analytical Luminescence (San Diego, CA, USA).

### *Cell culture*

The human ovarian adenoserous carcinoma cell line 2008 (DiSaia *et al.*, 1972) was carried as an exponentially growing monolayer in a humidified incubator at 37°C and 5% carbon dioxide in RPMI-1640 supplemented with 5% fetal calf serum and 2 mM glutamine.

### *Luciferase assay*

pGADD-LUC, a *GADD153* promoter-driven luciferase reporter construct was created by ligating the *ClaI*/*Hin*dIII fragment of p9000 (a gift from Dr NJ Holbrook, NIA, NIH, Baltimore, MD, USA), containing the hamster *GADD153* promoter into the *AccI*/*Hin*dIII site of pB-LUC (Luethy *et al.*, 1990). pB-LUC contains the firefly luciferase gene ligated into the *Bam*HI site of pBluescript $KS^-$ (a gift from Dr L Quattrochi).

The cells were transfected with the pGADD-LUC construct by a modification of the method described by Rose *et al.* (1991). Cells were plated at $3 \times 10^5$ cells per 35 mm dish, and then 18 h later they were incubated at 37°C with 5 μg of plasmid DNA and 30 μl of liposomes in 1 ml of RPMI-1640. After 3 h the lipids were removed and the cells were treated with cDDP for 1 h or taxol for 24 h. Six hours after the end of cDDP exposure, or after the 24 h taxol exposure, the cells were lysed in 500 μl of lysis buffer (25 mM glycylglycine, pH 7.8, 15 mM magnesium sulphate, 4 mM EGTA, 1% Triton X-100, 1 mM dithiothreitol). Luciferase activity was measured by a modification of the method described by Brasier *et al.* (1989). Cell lysate (50 μl) was added to 200 μl of reaction buffer (lysis buffer with 15 mM potassium phosphate, pH 7.8, and 2 mM ATP added). Light emission was measured after injection of 100 μl of 1 mM luciferin into the lysate/reaction mixture using a MonoLight 2001 (Analytical Luminescence).

### *Northern blotting*

Total cellular RNA was extracted and Northern blots prepared using MagnaGraph nylon membranes (MSI, Westboro, MA, USA) by standard techniques (Davis *et al.*, 1986). The extent of hybridisation was quantified by the Molecular Imager System (Bio-Rad, Hercules, CA, USA). The human *GADD153* probe was a gift of Dr NJ Holbrook. Lane loading differences were corrected for by comparison to the same blot hybridised with a β-actin probe.

### *Colony forming assays and median effect analysis*

Three hundred cells were plated per dish and allowed to attach overnight. The cells were treated with tyrphostin B46 or DMSO for 1 h followed by a 1 h concurrent exposure with DDP, or a concurrent 24 h exposure to taxol. After DDP exposure, tyrphostin B46 or DMSO was added back to the media for a total exposure of 24 h. After drug exposure, the media was replaced and at 10 days after treatment, colonies of 50 cells or more were counted. Median effect analysis was carried out as described by Chou and Talalay (1986).

### Results

### *Effect of taxol and cDDP on activation of the* GADD153 *promoter and endogenous* GADD153 *mRNA*

Human ovarian 2008 cells were transiently transfected with pGADD-LUC, which contains 786 basepairs of the hamster *GADD153* promoter coupled to the luciferase reporter gene. They were then exposed to taxol for 24 h over a concentration range corresponding to 1–10 times the $IC_{50}$, and luciferase activity was measured 24 h after the start of drug exposure, which previous work had shown to be the time of peak luciferase activity (data not shown). Figure 1a shows the change in luciferase activity expressed relative to the level in untreated control cells; and indicates that taxol activated the *GADD153* promoter in a concentration-dependent manner. Figure 1b shows the effect of the same taxol exposure on the change in endogenous *GADD153* mRNA levels in non-transfected cells determined by Northern blot analysis of RNA harvested 24 h after the start of drug exposure. Taxol increased the level of endogenous *GADD153* mRNA in a dose-dependent manner that corresponded well with its effect on *GADD153* promoter activation.

**Figure 1** Effect of taxol on the activation of the *GADD153* promoter and on endogenous *GADD153* mRNA levels. (**a**) Luciferase activity measured in cells transiently transfected with pGADD-LUC. (**b**) Fold change in endogenous gadd153 mRNA levels quantified by Northern analysis normalised for β-actin expression. In both types of experiments, measurements were made 24 h after the start of taxol exposure. Bars represent the mean of two (**b**) or three (**a**) experiments performed with duplicate cultures. Vertical bars ± s.e.

Similar experiments were conducted with cDDP in which 2008 cells were transiently transfected with pGADD-LUC and exposed to cDDP for 1 h. Maximal levels of luciferase activity were found to occur at 6 h after the end of a 1 h drug exposure (data not shown). Figure 2a shows that over an equitoxic concentration range cDDP caused substantially less activation of the *GADD153* promoter, as reflected by luciferase activity, than did taxol. However, Figure 2b shows that cDDP produced up to a 40-fold increase in endogenous *GADD153* mRNA level after drug treatment (note the difference in the ordinate scale in a and b). Thus, in contrast to what was observed with taxol, in the case of cDDP there was a much smaller effect on promter activity than on endogenous *GADD153* level.

Figure 3 shows that there is a good correlation between the fold change in endogenous mRNA and the fold activation of the *GADD153* promoter for cDDP ($r = 0.94$) and taxol ($r = 0.96$). However the slope of the least mean squares line was 1.1 for taxol as opposed to 29.5 for cDDP. Thus, for a given degree of activation of the transfected promoter, the effect on endogenous *GADD153* mRNA level was 27-fold greater for cDDP than for taxol. This indicates that the mechanisms by which the two drugs produce these changes differ in at least some components.

Figure 4 shows an analysis of the correlation between the degree of cytotoxicity produced by the drug exposure and the change in endogenous *GADD153* mRNA. For both agents there was an excellent correlation (cDDP, $r = 0.98$; taxol, $r = 0.97$). As cell kill was increased with cDDP there was a much greater effect on *GADD153* mRNA levels than when cell kill was increased with incrementally higher concentrations of taxol. This indicates that *GADD153* mRNA levels did not vary solely as a function of the degree of toxicity, but were also a function of the specific drug which caused the cell death.

*Effect of tyrphostin B46 on GADD153 promoter activation*

Tyrphostins are inhibitors of tyrosine kinases that were originally designed to compete for the substrate binding site of the epidermal growth factor receptor tyrosine kinase. Gazit *et al.* (1989, 1991) found that tyrphostin B46 can inhibit epidermal growth factor-induced proliferation with an $IC_{50}$ of 2.5 μM. We compared the ability of tyrphostin B46 to alter the taxol and cDDP-induced increase in luciferase activity following transfection of pGADD-LUC into 2008 cells. After transfection, cells were treated withl0 μM tyrphostin B46 for 1 h and then concurrently with 70 nM taxol and tyrphostin B46 for 24 h. The data presented in Figure 5a shows that, relative to the vehicle alone control, 10 μM tyrphostin B46 had no significant effect on the basal activity of the pGADD-LUC, nor on the ability of taxol to activate the *GADD153* promoter. In contrast, tyrphostin B46 was able to inhibit the response elicited by a 1 h exposure to cDDP. As shown in Figure 5b, 50 μM DDP induced a 2.6-fold increase in luciferase activity 6 h after exposure compared with untreated controls. Treatment with tyrphostin B46 for 7 h had

**Figure 2** Effect of cDDP on the activation of the *GADD153* promoter and on endogenous *GADD153* mRNA levels. (**a**) Luciferase activity measured in cells transiently transfected with pGADD-LUC 6 h after a 1 h treatment with cDDP. (**b**) Fold change in endogenous *GADD153* mRNA levels quantified by Northern analysis normalised for β-actin expression. Total RNA was extracted 24 h after a 1 h cDDP exposure. Bars represent the mean of two (**b**) or three (**a**) experiments performed with duplicate cultures. Vertical bars ± s.e.

**Figure 3** Correlation between drug effect on relative endogenous *GADD153* mRNA levels and relative luciferase activity. For known doses of drug, endogenous mRNA levels were plotted relative to luciferase activity. Linear regression curves (linear least squares method) were plotted using DeltaGraph for the Macintosh software (DeltaPoint, Monterey, CA, USA). ■, Taxol; ●, cDDP.

**Figure 4** Correlation between drug effect on relative endogenous *GADD153* mRNA levels and relative cytotoxicity as measured by $IC_{50}$. For known doses of drug, endogenous mRNA levels were plotted relative to fold $IC_{50}$. Linear regression curves (linear least squares method) were plotted using DeltaGraph for the Macintosh software (DeltaPoint). ■, Taxol; ●, cDDP.

no significant effect on the basal activity of the *GADD153* promoter. However, tyrphostin B46 significantly inhibited the cDDP-induced activation of the *GADD153* promoter in a dose-dependent manner, ($P = 0.009$ at 1 μM and $P < 0.0001$ at 10 μM). Treatment with 1 μM genistein, another modulator of tyrosine kinases, had no effect on the ability of either taxol or cDDP to increase luciferase activity (data not shown). Thus, the signal transduction pathways leading to activation of the *GADD153* promoter by cDDP and taxol differ at least with respect to the extent of involvement of a tyrphostin inhibitable kinase.

**Figure 5** The effect of tyrphostin B46 on the induction of *GADD153* promoter activity by taxol (**a**) and cDDP (**b**). Luciferase activity is expressed relative to untreated control cells. ■, Control treated cells; □, 70 nM taxol-treated cells (**a**) or 50 μM cDDP-treated cells (**b**) ± s.d. *, $P = 0.0009$. **, $P < 0.0001$.

*Effect of tyrphostin B46 on the toxicity of cDDP and taxol*

In order to investigate the effect of *GADD153* promoter activation on the toxicity of cDDP and taxol in the 2008 cells, we performed colony-forming assays. The cells were allowed to attach overnight, exposed to 10 μM tyrphostin B46 for 1 h and then concurrently with cDDP (1 h) or taxol (24 h). As shown in Figure 6, incubation with 10 μM tyrphostin B46 decreased the $IC_{50}$ of cisplatin 2.3-fold from 5.0 μM cDDP to 2.2 μM cDDP ($P = 0.013$, *t*-test). Since tyrphostin B46 is slightly toxic (10 μM is an $IC_5$), one cannot determine whether the interaction with cDDP is additive, antagonistic or synergistic simply by subtraction. In order to determine the nature of this interaction, the toxicity of cDDP and tyrphostin B46 were investigated using median effect analysis (Chou and Talalay, 1986). Median effect analysis is a mathematically formal method of determining synergy, additivity or antagonism. If the combination index for the two drugs is equal to 1, the effect is additive, if the index is above 1 the effect is antagonistic, and if the combination index is below 1 the effect is synergistic. As shown in Figure 7, the majority of the combination index curve for cDDP and tyrphostin B46 falls below 1, indicating that the effect of the drugs is synergistic.

Since tyrphostin B46 decreased the activation of the *GADD153* promoter after cDDP exposure and also increased cDDP cytotoxicity, we investigated the effect of tyrphostin B46 on the cytotoxicity of taxol, in which tyrphostin had no effect on the activation of the *GADD153* promoter. As shown in Figure 8, tyrphostin had no significant effect on the

**Figure 7** Plot of the combination index as a function of cell kill for the interaction between cDDP and tyrphostin B46 against the 2008 cell line. Each data point represents the mean of three experiments performed in triplicate ± s.d.

**Figure 6** The effect of 10 μM tyrphostin B46 on the cytotoxicity of cDDP in the 2008 cell line. ■, Control treatment; ●, 10 μM tyrphostin B46. Each data point represents the mean of three experiments performed in triplicate ± s.d.

**Figure 8** The effect of 10 μM tyrphostin B46 on the cytotoxicity of taxol in the 2008 cell line. ■, control treatment; ●, 10 μM tyrphostin B46. Each data point represents the mean of three experiments performed in triplicate ± s.d.

cytotoxicity of taxol. In fact, there is a slight decrease in cytotoxicity at high concentrations of taxol.

### Discussion

The data presented in this report show that the activation of the *GADD153* promoter can occur through at least two distinct pathways, here defined by cDDP and taxol. There are three major pieces of evidence to suggest that two pathways exist (1) the relationship between activation of the promoter and increase in endogenous *GADD153* RNA is different for the two drugs; (2) the two drugs also differ in the relationship between cytotoxicity and change in endogenous *GADD153* RNA levels; (3) the promoter activation pathways are differentially inhibitable by tyrphostin B46.

The magnitude of the activation of the *GADD153* promoter corresponds closely to the change produced in endogenous mRNA level in response to activation of the taxol-inducible pathway (slope 1.1), but this relation is quite different for the pathway activated by cDDP (slope 29.5). Stated another way, for a given degree of activation of the transfected *GADD153* promoter, cDDP produced a much greater increase in endogenous *GADD153* mRNA than taxol. The ability of cDDP to activate the transfected *GADD153* promoter is rather weak. Based on results obtained with the promoter construct used in this study, the effect of cDDP on endogenous *GADD153* levels is likely to be mediated by elements other than those represented by the promoter elements present in pGADD-LUC. Alternative mechanisms such as stabilisation of the *GADD153* message may also play an important role in increasing *GADD153* mRNA levels in response to either drug. Jackman *et al.* (1994) have reported that DNA-damaging drugs will stabilise *GADD153* RNA and increase mRNA half-life, yet agents that cause cell cycle arrest (such as serum starvation and prostaglandin $A_2$) do not increase mRNA half-life. Our results are consistent with these findings. However, while we cannot exclude the possibility that increased mRNA stability accounts for the 26-fold difference between the increase in mRNA levels and promoter activity after cDDP-treatment, it seems more likely that the endogenous *GADD153* gene contains promoter elements that are not contained in pGADD-LUC. It is probable that there are other response elements either 5′, 3′ or within the introns of *GADD153*. In the case of *GADD45*, another member of the DNA damage-inducible gene family, a p53-responsive element is known to lie within the third intron (Kastan *et al.*, 1992; Hollander *et al.*, 1993).

The second piece of evidence indicating that cDDP and taxol activate *GADD153* expression by different mechanisms comes from the difference in the magnitude of the increase in *GADD153* mRNA produced by equitoxic exposures to the two drugs. We have shown that equitoxic schedules of exposure to the same drug produce equal increases in *GADD153* mRNA (Gately *et al.*, 1994). However, equitoxic exposures to cDDP and taxol produced quite different changes in the *GADD153* message. For example, at an exposure of twice the $IC_{50}$ (10 $\mu$M for cDDP, 10 nM for taxol), cDDP increased the *GADD153* message levels by 10-fold whereas taxol increased the message level by only 2-fold. The reason for this difference in message increase is unclear at this time. However, it may indicate that *GADD153* mRNA levels are more strongly increased by activation of the cellular injury response by an agent that directly causes DNA damage than by an agent that interferes with tubulin function. Further study is needed to address this question.

The third piece of evidence arguing for multiple pathways is the fact that activation of the *GADD153* promoter by taxol and cDDP is differentially inhibited by the tyrosine kinase inhibitor tyrphostin B46. To the extent that tyrphostin B46 is specific for tyrosine kinases, these data establish that such a kinase participates in the cDDP-inducible but not the taxol-inducible pathway. The conclusion that the cDDP activation pathway is different from that utilised by other agents is supported further by the observations of Luethy and Holbrook (1994), who found that tyrosine kinase inhibitors were also not able to block the increase in *GADD153* mRNA levels produced by methyl methanesulphonate and UV radiation. This suggests that taxol may be triggering the same pathway as methyl methanesulphonate and UV radiation. Currently there is no information about which tyrosine kinase might be playing a role in the cDDP-activated pathway. Devary *et al.* (1992) reported that the activation of the c-*jun* promoter after UV irradiation was dependent on the SRC tyrosine kinase pathway. SRC kinase activates the c-*jun* promoter by increasing AP-1 activity. This is unlikely to be the case for GADD153. While the *GADD153* promoter contains an AP-1 responsive site, it is not inducible by the phorbol ester TPA which increases AP-1 activity (Fornace *et al.*, 1988). This suggests that AP-1 activity is not responsible for increases in *GADD153* promoter activity after damage. In fact, co-transfection of pGADD-LUC with plasmids expressing c-*jun* or constitutively active c-*src* do not increase luciferase activity measured twenty-four hours after transfection (DP Gately, unpublished data).

The synergistic enhancement of cDDP cytotoxicity by tyrphostin B46 indicates that there is a tyrosine kinase activated by cDDP treatment that functions to protect the cell after damage. Inhibition of this tyrosine kinase increases cytotoxicity of cDDP. This kinase is not activated after taxol treatment, therefore inhibition by tyrphostin has no effect on the cytotoxicity of taxol. One of the downstream effects of this signal transduction cascade is the activation of the *GADD153* promoter. At this point, we cannot determine whether *GADD153* promoter activation is itself protective, or simply activated by a signalling cascade that also functions to protect the cell by other mechanisms. Recent evidence reported by Barone *et al.* (1994) indicates that GADD153 protein microinjected into cells causes $G_1$ arrest. Thus, *GADD153* may play a role in the $G_1$ checkpoint mechanism that is activated by cellular injury. This checkpoint is thought to allow cells to repair any DNA damage before entering the next S-phase. Our data fit well with this hypothesis as we have previously shown that *GADD153* mRNA levels correlate with cellular injury as measured using a colony forming assay (Gately *et al.*, 1994). As the degree of damage increases, the cell should increase the levels of protective genes (i.e. *GADD153*). If this increase is blocked (for example, by tyrphostin B46), the cell would be expected to be more sensitive to the injury. In the absence of agents that block *GADD153* promoter activity, increases in *GADD153* mRNA levels should still be a good marker of tumour injury.

Treatments that produce cellular injury activate a number of different pathways that may be involved in the cellular response to damage (Kramer *et al.*, 1990; Bartlett *et al.*, 1992; Devary *et al.*, 1992, 1993; Zhan *et al.*, 1993). However, what role each of these responses plays in cell survival is still unknown. Our conclusion that there exist multiple pathways that can influence *GADD153* expression is consistent with the *GADD153* promoter being a convergence point for several independent signal transduction pathways uniquely involved in the detection of different kinds of cellular injury.

**Abbreviations:**
cDDP, cisplatin; gadd, growth arrest and DNA damage; $IC_{50}$, concentration required to inhibit colony formation by 50%; TPA, 12-*O*-tetradecanoylphorbol-13-acetate; C/EBP, CAAT/enhancer-binding protein: LAP, liver-enriched transcriptional activator protein.

**Acknowledgements**
This work was supported in part by grant DHP-26 from the American Cancer Society, grant CA 55725 from the National Institutes of Health and grant CTR 4154 from the Council for Tobacco Research. This work was conducted in part by the Clayton Foundation for Research – California Division. Drs Christen and Howell are Clayton Foundation Investigators. Contributions to this work by DP Gately are in partial fulfillment of the PhD requirements in the Department of Biomedical Sciences. Portions of this work were presented at the Eighty-fifth Annual Meeting of the American Association for Cancer Research and the Keystone Symposia on the Molecular Basis of Cancer Therapy.

### References

ÅMAN P, RON D, MANDAHL N, FIORETOS T, HEIM S, ARHEDEN K, WILLEN H, RYDHOLM A AND MITELMAN F. (1992). Rearrangement of the transcription factor gene *CHOP* in myxoid liposarcomas with t(12;16)(q13;p11). *Genes Chrom. Cancer*, **5**, 278–285.

BARLETT JD, LUETHY JD, CARLSON SG, SOLLOTT SJ AND HOLBROOK NJ. (1992). Calcium ionophore A23187 induces expression of the growth arrest and DNA damage inducible CCAAT/enhancer-binding protein (C/EBP)-related gene *gadd153*. *J. Biol. Chem.*, **267**, 20465–20470.

BARONE MV, CROZAT A, TABAEE A, PHILIPSON L AND RON D. (1994). CHOP (GADD153) and its oncogenic variant, TLS-CHOP, have opposing effects on the induction of $G_1/S$ arrest. *Genes Dev.*, **8**, 453–464.

BRASIER AR, TATE JE AND HABENER JF. (1989). Optimized use of the firefly luciferase assay as a reporter gene in mammalian cell lines. *BioTechniques*, **7**, 1116–1122.

CHEN Q, YU K, HOLBROOK NJ AND STEVENS JL. (1992). Activation of the growth arrest and DNA damage-inducible gene *gadd 153* by nephrotoxic cysteine conjugates and dithiothreitol. *J. Biol. Chem.*, **267**, 8207–8212.

CHOU T-C AND TALALAY P. (1986). Quantitative analysis of dose–effect relationships: the combined effects of multiple drugs or enzyme inhibitors. *Adv. Enzyme Regul.*, **22**, 27–55.

CROZAT A, ÅMAN P AND RON D. (1993). Fusion of CHOP to a novel RNA-binding protein in human myxoid liposarcoma. *Nature*, **363**, 640–644.

DAVIS LG, DIBNER MD AND BATTEY JF. (1986). *Basic Methods in Molecular Biology*. Elsevier Science: New York.

DEVARY Y, GOTTLIEB RA, SMEAL T AND KARIN M. (1992). The mammalian ultraviolet response is triggered by activation of Src tyrosine kinase. *Cell*, **71**, 1081–1091.

DEVARY Y, ROSETTE C, DIDONATO JA AND KARIN M. (1993). NF-kB activation by ultraviolet light not dependent on a nuclear signal. *Science*, **261**, 1442–1445.

DISAIA PJ, SINKOVICS JG, RUTLEDGE FN AND SMITH JP. (1972). Cell-mediated immunity to human malignant cells. *Am. J. Obstet. Gynecol.*, **114**, 979–989.

FORNACE AJ, ALAMO I AND HOLLANDER MC. (1988). DNA damage-inducible transcripts in mammalian cells. *Proc. Natl Acad. Sci. USA*, **85**, 8800–8804.

FORNACE AJ, NEBERT DW, HOLLANDER C, LUETHY JD, PAPATHANASIOU M, FARGNOLI J AND HOLBROOK N. (1989). Mammalian genes coordinately regulated by growth arrest signal and DNA damaging agents. *Mol. Cell. Biol.*, **9**, 4196–4203.

FORNACE AJ, JACKMAN J, HOLLANDER MC, HOFFMAN-LIEBERMANN B AND LIEBERMANN DA. (1992). Genotoxic-stress-response genes and growth-arrest genes. *Ann. NY Acad. Sci.*, 139–153.

GATELY DP, JONES JA, CHRISTEN RC, BARTON RB, LOS G AND HOWELL SB. (1994). Induction of the growth arrest and DNA damage inducible gene *GADD153* by cisplatin *in vitro* and *in vivo*. *Br. J. Cancer*, **70**, 1102–1106.

GAZIT A, YAISH P, GILON C AND LEVITZKI A. (1989). Tyrphostins I : Synthesis and biological activity of protein tyrosine kinase inhibitors. *J. Med. Chem.*, **32**, 2344–2352.

GAZIT A, OHEROV N, POSNER I, YAISH P, GILON C AND LEVITZKI A. (1991). Tyrphostins 2: Heterocyclic and a-substituted benzylidenemalononitrile tyrphostins as potent inhibitors of EGF receptor and ErbB2/neu tyrosine kinases. *J. Med. Chem.*, **34**, 1896–1907.

HOLBROOK NJ AND FORNACE AJ. (1991). Response to adversity: molecular control of gene activation following genotoxic stress. *New Biol.*, **3**, 825–833.

HOLLANDER MC, ALAMO I, JACKMAN J, WANG MG, MCBRIDE W AND FORNACE AJ. (1993). Analysis of the mammalian *gadd45* gene and its response to DNA damage. *J. Biol. Chem.*, **32**, 24385–24393.

JACKMAN J, ALAMO I AND FORNACE AJ. (1994). Genotoxic stress confers preferential and coordinate messenger RNA stability on the five *gadd* genes. *Cancer Res.*, **54**, 5656–5662.

KASTAN MB, ZHAN Q, EL-DIERY WS, CARRIER F, JACKS T, WALSH WV, PLANKETT BS, VOGELSTEIN B AND FORNACE AJ. (1992). A mammalian cell cycle checkpoint pathway utilizing p53 and *GADD45* is defective in ataxia-telangiectasia. *Cell*, **71**, 587–597.

KRAMER M, STEIN B, MAI S, KONIG H, LOFERER H, GRUNICKE HH, PONTA H, HERRLICH P AND RAHMSDORF HJ. (1990). Radiation-induced activation of transcription factors in mammalian cells. *Radiat. Environ. Biophys.*, **29**, 303–313.

LUETHY JD AND HOLBROOK NJ. (1992). Activation of the *gadd 153* promoter by genotoxic agents: a rapid and specific response to DNA damage. *Cancer Res.*, **52**, 5–10.

LOUTHY JD AND HOLBROOK NJ. (1994). The pathway regulating *GADD153* induction in response to DNA damage is independent of protein kinase C and tyrosine kinases. *Cancer Res.*, **54**, 1902s–1906s.

LUETHY JD, FARGNOLI J, PARK JS, FORNACE AJ AND HOLBROOK NJ. (1990). Isolation and characterization of the hamster *gadd 153* gene. *J. Biol. Chem.*, **265**, 16521–16526.

PARK JS, LUETHY JD, WANG MG, FARGNOLI J, FORNACE AJ, MCBRIDE OW AND HOLBROOK NJ. (1992). Isolation, characterization and chromosomal localization of the human *GADD153* gene. *Gene*, **116**, 259–267.

PINTO AL AND LIPPARD SJ. (1985). Binding of the antitumor drug *cis*-diamminedichloroplatinum(II) (cisplatin) to DNA. *Biochim. Biophys. Acta*, **780**, 167–180.

PRICE BD AND CALDERWOOD SK. (1992). Gadd45 and Gadd153 messenger RNA levels are increased during hypoxia and exposure of cells to agents which elevate the levels of glucose-related proteins. *Cancer Res.*, **52**, 3814–3817.

RON D AND HABENER JF. (1992). CHOP, a novel developmentally regulated nuclear protein that dimerizes with transcription factors C/EBP and LAP and functions as a dominant negative inhibitor of gene transcription. *Genes Dev.*, **6**, 439–453.

ROSE JK, BUONOCORE L AND WHITT MA. (1991). A new cationic liposome reagent mediating nearly quantitative transfection of animal cells. *Biotechniques*, **10**, 520–525.

ROWINSKY EK, MCGUIRE WP AND DONEHOWER RC. (1993). The current status of taxol. In *Principles and Practice of Gynecologic Oncology Updates*, Hoskins WJ, Perez CA and Young RC. (eds). pp. 1–16. JB Lippincott: Philadelphia.

ROWLEY R, HUDSON J AND YOUNG PG. (1992). The *weel* protein kinase is required for radiation-induced mitotic delay. *Nature*, **356**, 353–355.

WALKER GC. (1985). Inducible DNA repair systems. *Annu. Rev. Biochem.*, **54**, 425–457.

WEINERT TA AND HARTWELL LH, (1988). The *RAD9* gene controls the cell cycle response to DNA damage in *Saccharomyces cervisiae*. *Science*, **241**, 317–322.

ZHAN Q, CARRIER F AND FORNACE AJ. (1993). Induction of cellular p53 activity by DNA-damaging agents and growth arrest. *Mol. Cell. Biol.*, **13**, 4242–4250.